# Doxorubicin-induced modulation of TGF-β signaling cascade in mouse fibroblasts: insights into cardiotoxicity mechanisms

**DOI:** 10.1038/s41598-023-46216-7

**Published:** 2023-11-02

**Authors:** Conner Patricelli, Parker Lehmann, Julia Thom Oxford, Xinzhu Pu

**Affiliations:** 1 Biomolecular Sciences Graduate Programs, Boise State University, Boise, ID 83725-1512 USA; 2 Idaho College of Osteopathic Medicine, Meridian, ID 83642-8046 USA; 3 Biomolecular Research Center, Boise State University, Boise, ID 83725-1511 USA; 4 Department of Biological Sciences, Boise State University, Boise, ID 83725-1515 USA

**Keywords:** Biochemistry, Cancer, Medical research

## Abstract

Doxorubicin (DOX)-induced cardiotoxicity has been widely observed, yet the specific impact on cardiac fibroblasts is not fully understood. Additionally, the modulation of the transforming growth factor beta (TGF-β) signaling pathway by DOX remains to be fully elucidated. This study investigated DOX’s ability to modulate the expression of genes and proteins involved in the TGF-β signaling cascade in mouse fibroblasts from two sources by assessing the impact of DOX treatment on TGF-β inducible expression of pivotal genes and proteins within fibroblasts. Mouse embryonic fibroblasts (NIH3T3) and mouse primary cardiac fibroblasts (CFs) were treated with DOX in the presence of TGF-β1 to assess changes in protein levels by western blot and changes in mRNA levels by quantitative reverse transcriptase polymerase chain reaction (qRT-PCR). Our results revealed a dose-dependent reduction in cellular communication network factor 2 (CCN2) protein levels upon DOX treatment in both NIH3T3 and CFs, suggesting an antifibrotic activity by DOX in these fibroblasts. However, DOX only inhibited the TGF-β1 induced expression of COL1 in NIH3T3 cells but not in CFs. In addition, we observed that DOX treatment reduced the expression of BMP1 in NIH3T3 but not primary cardiac fibroblasts. No significant changes in SMAD2 protein expression and phosphorylation in either cells were observed after DOX treatment. Finally, DOX inhibited the expression of Atf4 gene and increased the expression of Cdkn1a, Id1, Id2, Runx1, Tgfb1, Inhba, Thbs1, Bmp1, and Stat1 genes in NIH3T3 cells but not CFs, indicating the potential for cell-specific responses to DOX and its modulation of the TGF-β signaling pathway.

## Introduction

Chemotherapy has improved cancer survival rates, however, side effects of chemotherapeutic drugs remain a concern. An increased risk of cardiovascular complications and cardiotoxicity is attributed to anthracyclines^1–4^. Anthracyclines are a class of naturally occurring antibiotics produced by Streptomyces bacteria that exhibit broad anticancer efficacy. One of the most widely used anthracyclines is DOX, which was approved by the FDA in 1974 for the treatment of cancers, including hematopoietic cancers such as leukemias and lymphomas, and metastatic solid tumors such as breast, gastric, neuroblastoma, lung, ovarian, and bladder cancers in both adult and pediatric patients^5^.

DOX has been known to damage various types of cells in the heart, including cardiomyocytes, fibroblasts, and endothelial cells, which can lead to lethal cardiomyopathy^6–9^. Echocardiography and cardiac magnetic resonance imaging are standard methods used to monitor the left ventricle ejection fraction (LVEF) to assess the progression of cardiotoxicity induced by DOX^10–14^. DOX-induced cardiotoxicity is characterized by a gradual decline in LVEF which, if not managed, can lead to heart failure. A significant reduction in LVEF is defined as a decrease of more than 10% from baseline to a value below the lower limit of normal (usually less than 50–55%)^14–16^. The risk of developing cardiotoxicity increases with the cumulative dose of DOX^17^. The reported risk of DOX-induced cardiotoxicity by cumulative dose is 3–5% for 400 mg/m^2^, 7–26% for 550 mg/m^2^, and 18–48% for 700 mg/m^2^^18–22^. Moreover, age is a significant factor that contributes to the susceptibility to cardiotoxicity in DOX-treated patients. Patients under five years old or over sixty-five years old are more vulnerable to developing cardiotoxicity^18,23^. It is likely that these age groups are more prone to cardiotoxicity due to age-related differences in the cardiovascular system which also increase the risk of developing heart failure.

DOX exerts its antitumor activity by intercalating into DNA, which inhibits the binding of topoisomerase IIβ and the unwinding of supercoiled DNA^24–27^. This inhibition leads to a blockage of DNA synthesis and the eventual induction of double-stranded DNA breaks, triggering programmed cell death in cells^28,29^. In addition, DOX has been shown to induce cellular senescence in tumor and normal cells^30–35^. Despite its therapeutic benefits, DOX is associated with off-target effects, which include the production of reactive oxygen species (ROS). The precise mechanisms underlying DOX-induced ROS production are not fully understood, however, studies suggest that mitochondrial dysfunction^36,37^, calcium homeostasis^38,39^, iron chelation^40,41^, and inflammatory responses^42–45^ are implicated in ROS generation caused by DOX. Although antioxidants have been used in combination with DOX to decrease the buildup of free radicals, this approach has not proven effective in reducing cardiotoxicity while maintaining the antitumor efficacy of DOX^46^.

The myocardium is a complex tissue comprising multiple cell types, including cardiac fibroblasts (CFs) and endothelial cells (ECs)^47^. CFs and ECs play crucial roles in regulating the expression and accumulation of extracellular matrix (ECM) components such as collagens, fibronectin, matrix metalloproteinases (MMPs), and endogenous protease inhibitors known as tissue inhibitors of metalloproteinases (TIMPs)^48–52^. Transforming growth factor β (TGF-β) is a pivotal cytokine/growth factor that has been shown to stimulate fibroblasts^53,54^. The TGF-β signaling pathway has been implicated in fibrosis in the myocardium, which contributes to cardiac dysfunction^55–59^. In this study, we investigated the effects of DOX on the TGF-β signaling cascade in mouse fibroblasts from two sources to better understand the mechanisms of its cardiotoxicity.

## Results

### Effect of DOX on fibroblast viability

**Figure 1:**
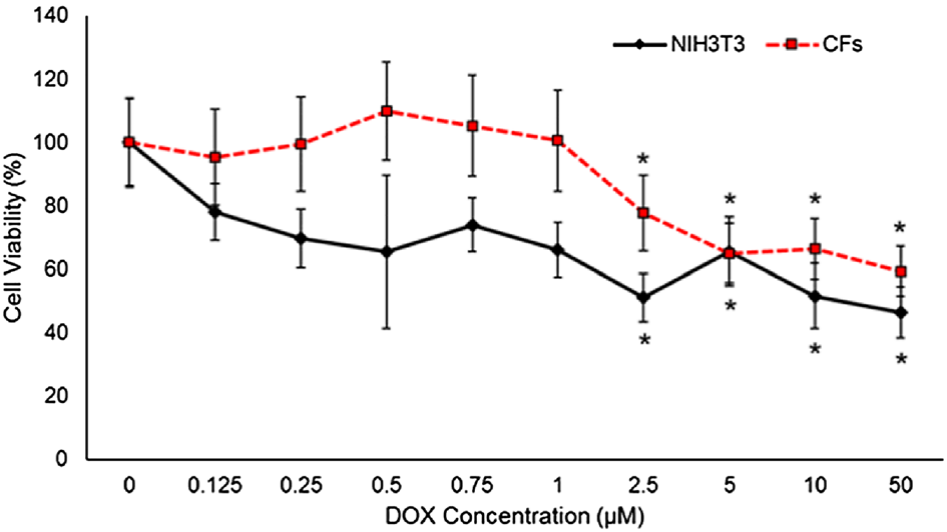
Cytotoxicity of DOX in primary cardiac fibroblast cells isolated form BALB/c mice and NIH3T3 embryonic fibroblast cell line. Cells were treated with series concentrations of DOX (0–50µM) for 24 h. Cell viability was assessed using the alamarBlue assay. Values represent mean ± standard error (n = 6). *P-value < 0.05 compared to control (Student’s t-test).

Cell viability of NIH3T3 and CFs was evaluated using an alamarBlue assay. Cells were treated with 0 – 50 µM DOX for 24 h. A significant decrease in cell viability was observed at 2.5 µM DOX and above (Fig. 1).

### DOX treatment Inhibited CCN2 protein expression in a dose-dependent manner

**Figure 2:**
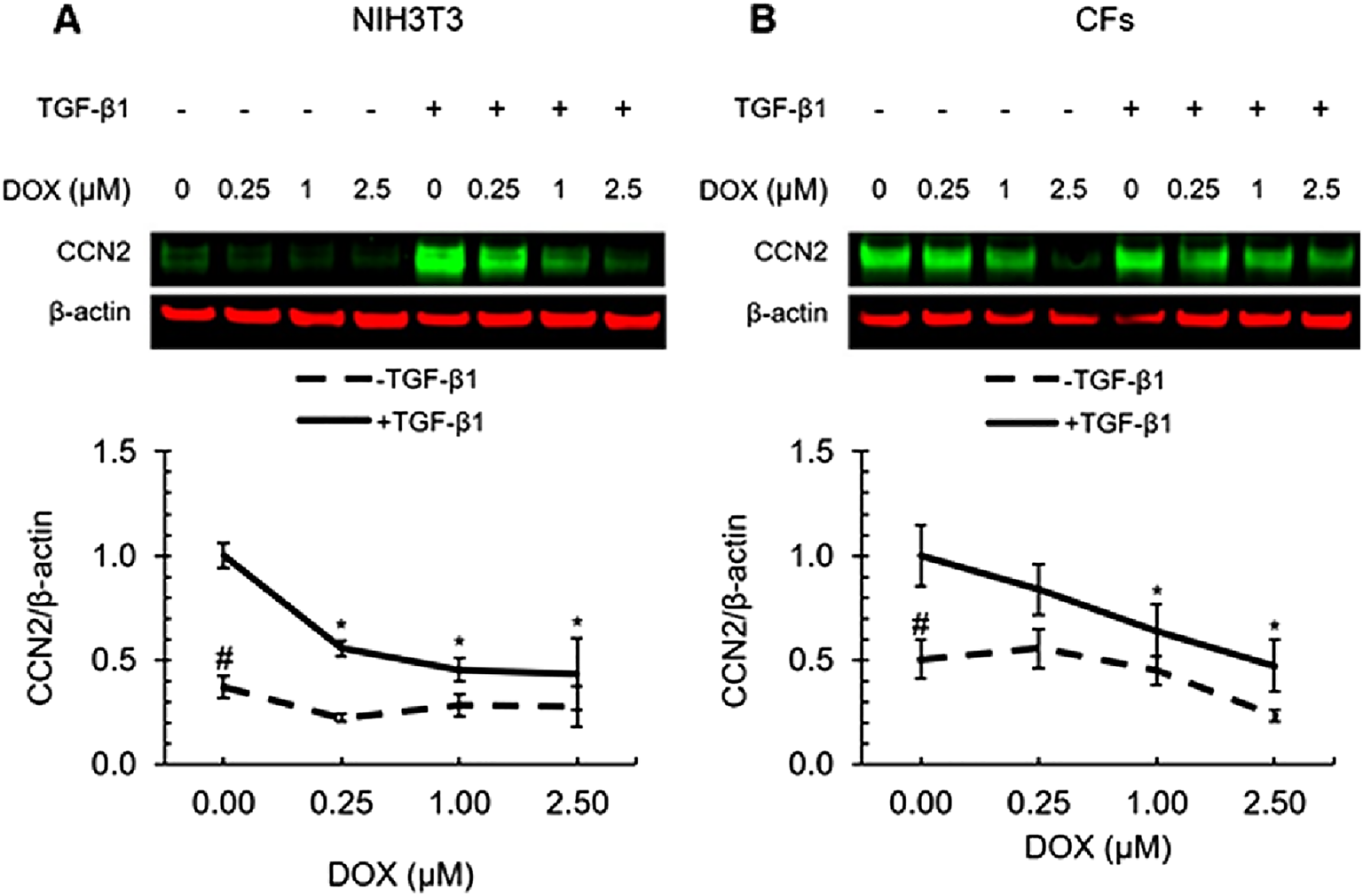
DOX inhibited CCN2 expression in a dose-dependent manner. Western blot analysis of the effects of DOX on CCN2 expression in NIH3T3 cells (**A**) and CFs (**B**). Densitometry results normalized to the treatment group with TGF-β1 and without DOX. Values represent mean ± standard error (n = 4). *P-value < 0.05 compared to treatment group containing TGF-β1 and lacking DOX (two-way ANOVA with Dunnett’s test). # *P*-value < 0.05 compared to treatment group containing TGF-β1 and lacking DOX (two-way ANOVA with Dunnett’s test).

Cellular communication network factor 2 (CCN2), formerly known as connective tissue growth factor (CTGF), is a matricellular protein involved in many cellular processes^64,65^. To assess the impact of DOX on CCN2 in fibroblasts, we employed western blot techniques to evaluate CCN2 protein levels in NIH3T3 cells and CFs. The cells were exposed to increasing concentrations of DOX (0, 0.25, 1, or 2.5 µM) in the presence and absence of TGF-β1 stimulation, for 24 h. CCN2 protein expression in both NIH3T3 and CFs was stimulated by TGF-β1 (Fig. 2). However, CFs showed a more robust constitutive expression of CCN2 than that of NIH3T3 in the absence of TGF-β1. A dose-dependent downregulation of CCN2 protein expression was observed in both cell lines treated for 24 h with DOX (Fig. 2). These results suggest that DOX modulates the expression of CCN2 in fibroblasts and that DOX may play a role in ECM remodeling.

### DOX treatment reduced the expression of BMP1 in NIH3T3 but not primary cardiac fibroblasts

**Figure 3:**
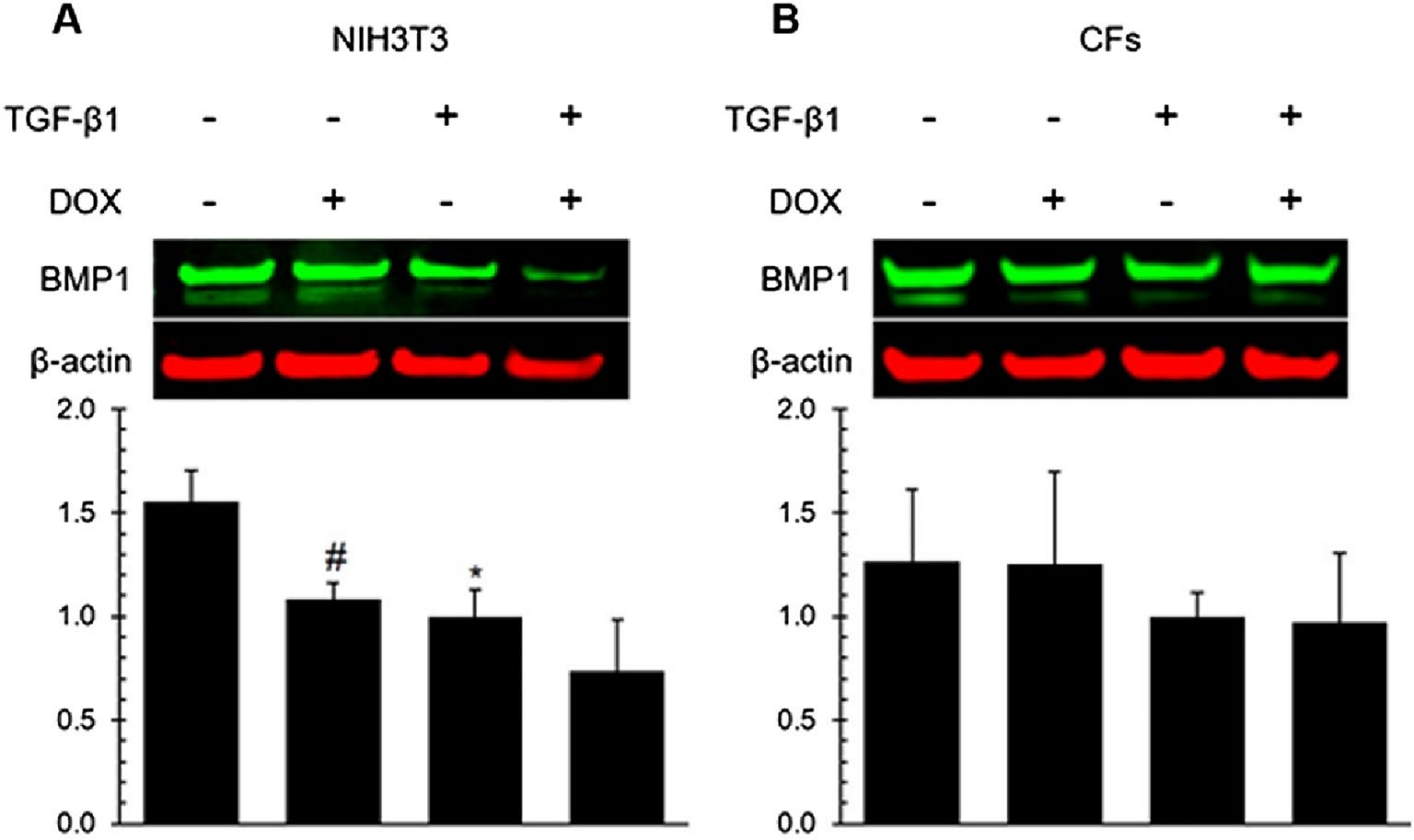
DOX significantly reduced BMP1 protein levels in NIH3T3 cells but not in CFs. Western blot analysis of the effect of DOX on BMP1 expression in NIH3T3 cells (**A**) and CFs (**B**). Densitometry results were normalized to the treatment group with TGF-β1 and without DOX. Values represent mean ± standard error (n = 3). * P-value < 0.05 compared to the treatment group with TGF-β1 and without DOX (Student’s t-test). # P-value < 0.05 compared to the treatment without TGF-β1 or DOX (Student’s t-test).

BMP1 (Bone Morphogenetic Protein 1), a pivotal member of the astacin family of metalloproteinases, plays a central role in processing and maturing extracellular matrix components, particularly collagens^88^. To investigate whether DOX’s potential impact on BMP1 expression in fibroblasts, cells were treated with DOX at a concentration of 1 µM for 24 h and BMP1 protein expression was assessed. DOX induced a significant decrease in BMP1 expression in NIH3T3 cells without the presence of TGF-β1, but not in CFs (Fig. 3). These results suggest that DOX suppresses BMP1 protein expression in a cell dependent manner.

### DOX did not induce the phosphorylation of SMAD2 in NIH3T3 cells and CFs

**Figure 4:**
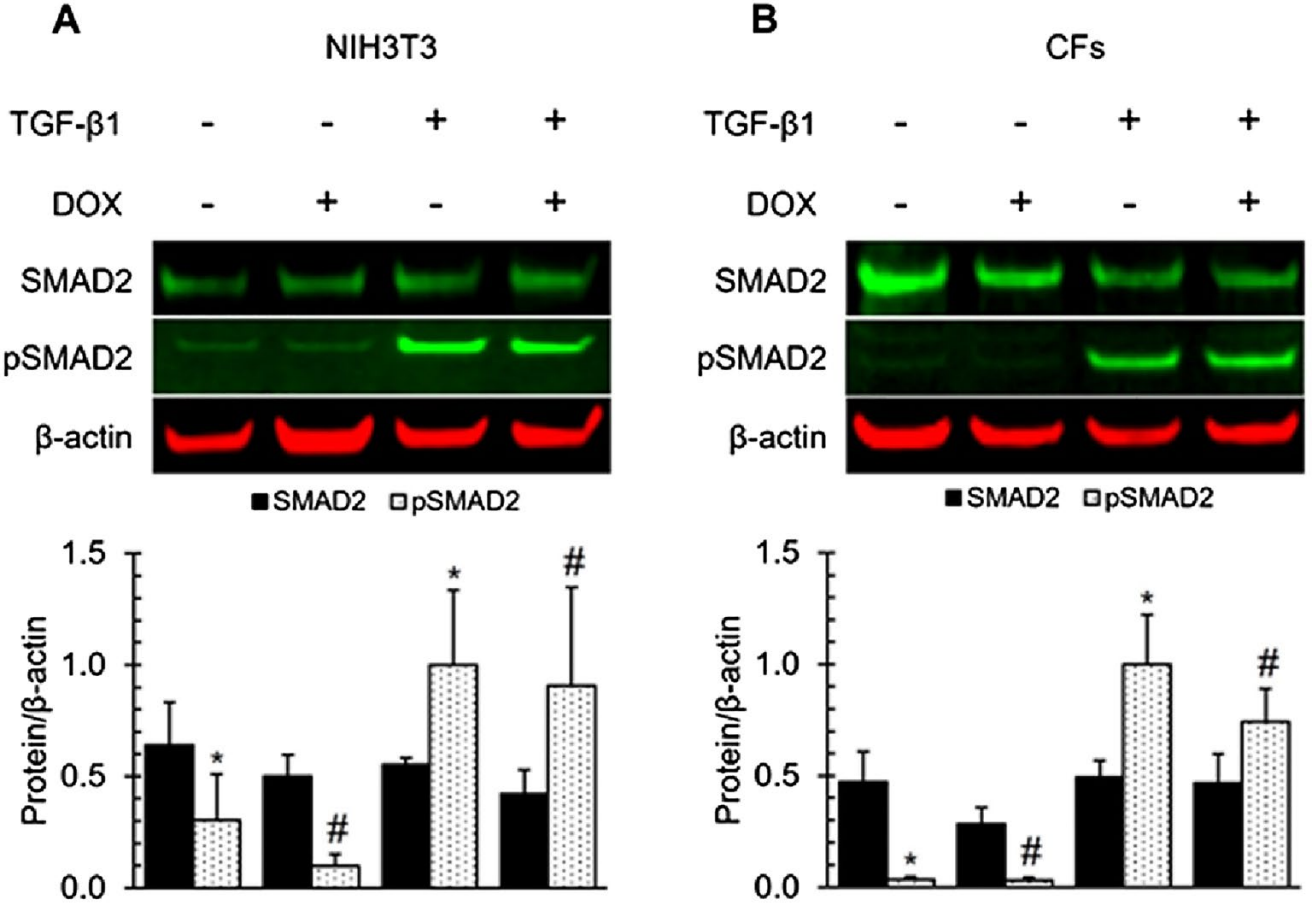
TGF-β1 induced the phosphorylation of SMAD2 in both NIH3T3 cells and CFs. DOX treatments did not significantly effect SMAD2 or pSMAD2 in one hour. Western blot analysis of NIH3T3 cells (**A**) and CFs (**B**) probing SMAD2 and phosphorylated SMAD2 . Densitometry was performed on SMAD2, pSMAD2, and normalized to housekeeping protein β-actin then normalized to pSMAD2 of the group treated with TGF-β1 and without DOX. Values represent mean ± standard error (n = 4).* *P*-value < 0.05 compared to normalized pSMAD2 (Student’s t-test). #, *P*-value < 0.05 compared to treatments with DOX and with and without TGF-β1 (Student’s t-test).

To investigate whether DOX affects the activation of the TGF-β/SMAD2 signaling pathway, we assessed SMAD2 and pSMAD2 in NIH3T3 and CFs treated with TGF-β1 and DOX for one hour. No significant changes in SMAD2 protein expression in either cells were observed after DOX treatment (Fig. 4). TGF-β1 stimulation led to a significant increase in pSMAD2 in both cell lines, in agreement with previous findings^61^. DOX didn’t induce significant changes in SMAD2 phosphorylation (Fig. 4).

### DOX treatment inhibited the TGF-β-induced COL1 protein expression in NIH3T3 cells but not CFs

**Figure 5:**
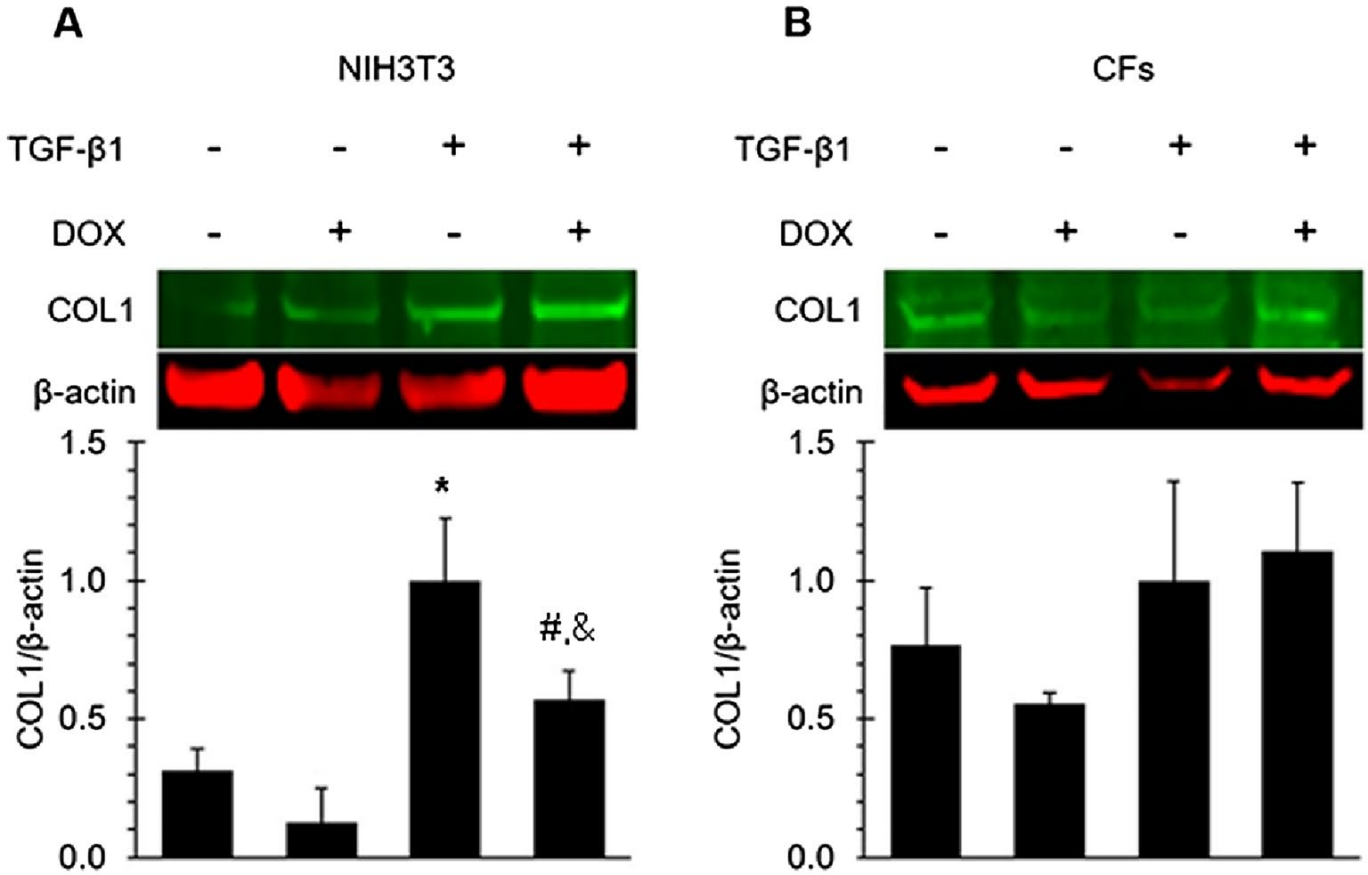
DOX inhibited the TGF-β1 induced expression of COL1 in NIH3T3 cells but not in CFs. Western blot analysis of NIH3T3 cells (**A**) and CFs (**B**) probing COL1 protein levels with and without TGF-β1 stimulation and DOX. Densitometry results were normalized to the treatment of TGF-β and without DOX. Values represent mean ± standard error (n = 4). *, *P*-value < 0.05 compared to treatment stimulated with TGF- β1 without DOX (Student’s t-test). #, *P*-value < 0.05 compared to treatment group stimulated with TGF-β1 and DOX (Student’s t-test).

Collagen Type I (COL1) is a fundamental structural protein essential for maintaining tissue integrity and is predominantly secreted by fibroblasts^57,85^. To assess the impact of DOX on COL1 protein expression in NIH3T3 cells and CFs, we conducted western blot analysis following a 24-h treatment with DOX and TGF-β1. Our results revealed that TGF-β1 treatment induced an increase in COL1 protein levels in NIH3T3 cells (Fig. 5). The presence of DOX effectively inhibited the TGF-β1-induced expression of COL1 protein (Fig. 5). These findings suggest that DOX treatment exerts a suppressive effect on COL1 production, which may have implications for the deposition of mature collagen within the extracellular matrix surrounding cardiac fibroblasts.

### DOX induced differential expression of genes in the TGF-β/BMP signaling pathway in NIH3T3 cells but not CFs

**Figure 6:**
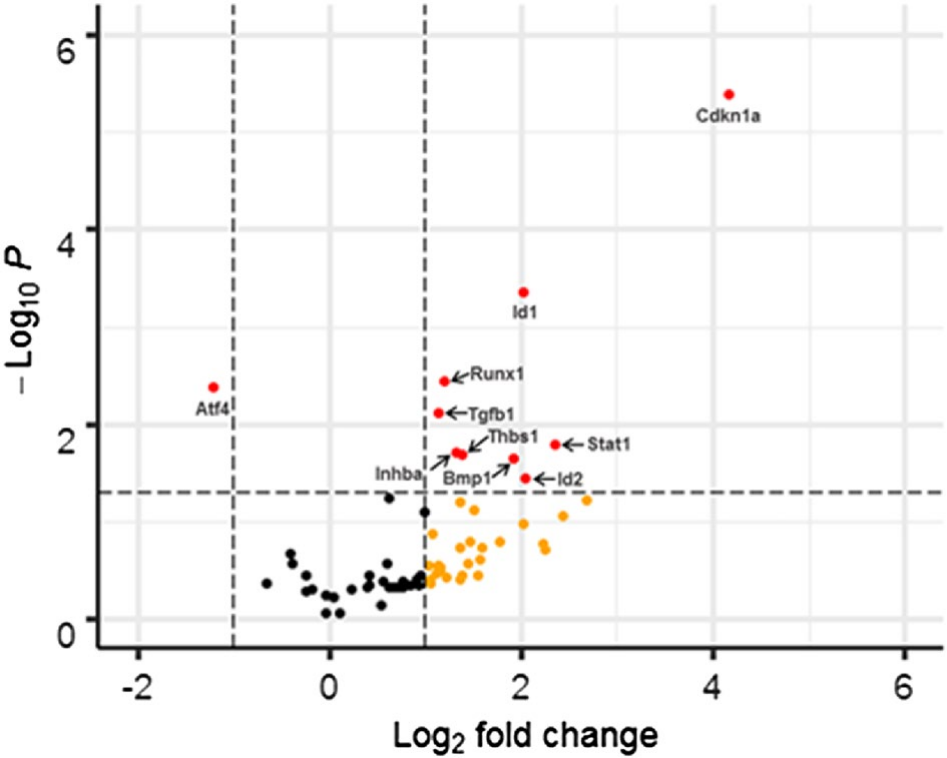
DOX inhibited the TGF-β1 induced expression of Atf4 and increased the expression of Cdkn1a, Id1, Id2, Runx1, Thbs1, Tgfb1, and Stat1 in NIH3T3 cells. Cells were treated with 10 ng TGF-β1 with or without 1 µM DOX for 24 h. Experiments were performed in triplicates. Student's t-test with Bonferroni correction was used for statistical analysis.

In this study, we examined the effects of DOX on the expression of genes in the TGF-β/BMP signaling pathway using qRT-PCR. DOX-induced differential expression of several genes in this pathway were observed in NIH3T3 cells, including upregulation of Id1, Id2, Runx1, Tgfb1, Inhba, Thbs1, Bmp1, and Stat1, and downregulation of Atf4 (Fig. 6, Table 1). In contrast, no significant gene alterations were observed in CFs under the same conditions (Supplementary Fig. 1). These findings indicate that DOX impacts TGF-β and BMP signaling in a cell type-specific manner.

**Table 1:** Up/Downregulated Genes in NIH3T3 Cells Treated with DOX and TGF-β1.

Gene symbol	Gene name	Alias	Regulation
Atf4	Activating transcription factor 4	Creb-2. Taxreb67, Txreb	Down
Bmp1	Bone morphogenetic protein 1	Bmp-1, Pcolc, Tolloid-like	Up
Cdkn1a	Cyclin dependent kinase inhibitor 1A	p21, Cap20, Cip1, Waf1, Sd1, p21cip1, cdkn1	Up
Id1	Inhibitor of DNA Binding 1	Bhlhb24	Up
Id2	Inhibitor of DNA Binding 2	Bhlhb26, Gig8	Up
Inhba	Inhibin subunit beta A	Edf, Activin beta-A chain	Up
Runx1	RUNX Family transcription factor 1	Amlcr1, Cbfa2, Aml1, Aml1/ETO, Pebp2a2	Up
Stat1	Signal transducer and activator of transcription 1	Stat91, Isgf-3	Up
Tgfb1	Transforming groth factor beta 1	Tgfbeta, Tgfb, Ced, Dpd1	Up
Thbs1	Thrombospondin 1	Tsp1, Tsp, Thbs-1, Tsp-1	Up

## Discussion

DOX is a widely used antibiotic with broad antitumor efficacy for treating hematopoietic and metastatic solid tumor cancers. However, its cardiotoxic side effect limits its clinical use. DOX-induced cardiotoxicity may manifest within hours of initial treatment and persist up to twenty years post-final administration, posing a potential lifelong risk for patients treated with DOX^17–22^. Despite five decades of research, there is still no therapeutic option to reduce the risk of developing cardiotoxicity while maintaining its antitumor efficacy. While cardiomyocytes are the cells within the myocardium responsible for the contraction of the heart, CFs, and vascular endothelial cells are responsible for the synthesis, deposition, and degradation of cardiac extracellular matrix (ECM) and play critical roles in maintaining normal functions of the heart^62,63^.

In this study, we investigated and compared the effects of DOX on TGF-β signaling cascade in primary cardiac fibroblasts and NIH3T3 embryonic fibroblasts. We first examined the protein expression of CCN2 in the cells following DOX treatment. A dose-dependent downregulation of CCN2 protein expression was observed in both cells treated for 24 h with DOX. CCN2 is highly expressed during the organ development stage and its expression is limited during adulthood^64,65^. The expression of CCN2 is greatly upregulated during the fibrogenic process, in which fibroblasts are activated^64,65^. CCN2 expression was minimal in NIH3T3 without the treatment of TGF-β1, a known stimulator of CCN2^64,66^. Interestingly, constitutive CCN2 was more robust in CFs, suggesting that CFs might have undergone activation. A similar observation was reported in primary hepatic stellate cells (HSCs)^67^. A strong expression of CCN2 in HSCs was found and TGF-β1 did not simulate CCN2 expression^67^. These data suggest that constitutive expression of CCN2 and stimulation by TGF-β1 may be cell-dependent.

CCN2 is a multifunctional matricellular protein involved in many biological processes, including ECM production, migration, differentiation, angiogenesis, and apoptosis^68–70^. It is a central mediator of tissue remodeling and fibrosis^71^. CCN2 is upregulated during tissue remodeling and fibrogenesis. One of the chronical manifestations of DOX-induced cardiomyopathy is fibrosis in the myocardium. However, in our study, DOX was found to decrease CCN2 expression in both cell lines, which implicates an antifibrotic activity. This contradiction may result from differences in the length of exposure and underlying cellular responses induced by DOX. Though the mechanisms of fibrotic changes induced by DOX are not fully understood, it is believed fibrosis is caused by the death of cardiomyocytes induced by DOX via various mechanisms, which include oxidative stress, topoisomerase inhibition, ferroptosis, cardiogenetics, mitochondrial bio-energetics, and autophagy modulation^72–80^. Direct regulation of fibrogenic signaling by DOX is not clear. In this study, cells were treated with DOX for 24 h. The observed decrease in CCN2 expression suggests that acute exposure may induce a different response in fibrotic signaling in fibroblasts. DOX has been reported to impair wound healing, reduce collagen production, and inhibit skin fibroblast proliferation^81–84^. We further examined whether DOX-induced downregulation of CCN2 affects the expression of Collagen type I, which is the most abundant type of collagen and is a major structural component of the cardiac ECM^85,86^. We observed that TGF-β1 stimulated a significant increase in COL1 protein level in NIH3T3 cells. DOX treatment significantly suppressed TGF-β1-induced COL1 upregulation. In contrast, similar effects were not observed in primary cardiac fibroblasts. Such differences between the two cells may result from distinct expression patterns of CNN2 in each cell. As discussed above, while CCN2 expression was minimal in NIH3T3 and was greatly stimulated by TGF-β1, it was strongly expressed in CFs without the presence of TGF-β1. This may have contributed to the different effects of DOX in the downstream expression of COL1.

BMP1 is involved in the regulation of TGF-β signaling pathway by cleaving the pro-domain of TGF-β precursors, which releases the active TGF-β ligand that then binds to its cell surface receptor complex and activates downstream SMAD proteins^87,88^. In this study, we observed that DOX induced a significant decrease in BMP1 expression in NIH3T3 cells, but not in CFs (Fig. 3). These results suggest that DOX suppress BMP1 protein expression in a cell dependent manner.

SMAD proteins play an important role in TGF-mediated ECM gene expression and regulation. Phosphorylation of cytoplasmic mediators belonging to the SMAD family is needed for signaling from TGF- β type I receptor to the nucleus after ligand activation^89^. In this study, we examined whether SMAD2 and BMP1 were involved in DOX-induced downregulation of CCN2 expression in fibroblasts primary cardiac fibroblast cells and NIH3T3 cells. TGF-β1 induced a significant increase in SMAD2 phosphorylation as expected^61^. DOX did not affect SMAD2 protein expression and phosphorylation. These results indicate that SMAD2 played no significant roles in DOX-induced CCN2 downregulation observed in this study.

In addition, this study examined the effects of DOX on gene transcription in the TGF-β and BMP signaling pathways. Significant alterations in the expression of 10 genes were observed in NIH3T3 cells treated with DOX and TGF-β1, however, no differential gene expression was observed in CFs under the same conditions. Specificly, we observed that activating transcription factor 4 (Atf4) was significantly downregulated in NIH3T3 cells treated with TGF-β1 and DOX compared to TGF-β1 treatment alone. Prior reports have indicated a positive correlation between ATF4 protein levels and the expression of COL1^90^. These findings suggest a potential association between decreased expression of the Atf4 gene and reduced COL1 protein expression. This is further evidenced by the increased gene expression of Inhibitor of DNA binding 1 (Id1) and Runt-related Transcription Factor 1 (Runx1). Id1 is an inhibitor of the TGF-β-induced collagen synthesis^91^. RUNX1 has been demonstrated in various cancer studies to inactivate the TGF-β1/SMAD signaling pathway which is also responsible for the deposition of collagen^92^.

Interestingly, there was an upregulation of Tgfb1, transforming growth factor beta 1, mRNA expression. This could be explained by the increase in Thrombospondin 1 (Thbs1) mRNA which promotes Tgfb1 mRNA expression in fibroblasts^93^.

Signal transducer and activator of transcription 1 (Stat1), a downstream target of the TGF-β signaling pathway, was upregulated in NIH3T3 cells upon treatment with DOX + TGF-β1 compared to TGF-β1 treatment alone. Stat1 has been observed to inhibit the myofibroblast phenotype in cells of fibroblastic origin in vivo^94^. This would suggest that DOX prevents the transformation of fibroblasts to myofibroblasts. Lastly, we observed a significant upregulation of cyclin-dependent kinase inhibitor 1A (Cdkn1a) (also known as p21) in NIH3T3 cells following DOX treatment. Cdkn1a is a senescence marker for oxidative stress that induces G2 arrest in the cell cycle in human fibroblasts^95–98^. DOX has been reported to induce a time-dependent increase of P21^cip1/waf1^ in cultured neonatal rat cardiomyocytes^35^. DOX has also been shown to increase P21^cip1/waf1^ gene and protein expression in mouse liver and kidney in vivo^99^.

In summary, the results from this study suggest that DOX treatment can modulate the expression of key genes involved in TGF-β signaling, ECM synthesis, and remodeling in fibroblasts in a cell origin dependently manner. Further investigations are warranted to elucidate the underlying mechanisms and functional implications of these observations.

## Materials and methods

### Cell culture

Cardiac fibroblasts (CFs) from BALB/c mice were purchased from Cell Biologics ( Chicago, Illinois, USA) and cultured in fibroblast medium provided by the vendor, which contained 0.5 mL fibroblasts growth factor (0.1% v/v), 0.5 mL hydrocortisone (0.1% v/v), 5 mL 100 × antibiotic–antimycotic solution, 5 mL of 200 mM L-Glutamine, and 10% (v/v) fetal bovine serum. NIH3T3 cells were purchased from the American Type Culture Collection (Manassas, Virginia, USA) and cultured in high glucose Dulbecco’s Modified Eagle Medium (DMEM; Thermo Fisher Scientific; 11965092) supplemented with 10% (v/v) fetal bovine serum (Atlanta Biologicals; S22660), 5 mL of 10 units/L penicillin, and 5 mL 10 mg/mL streptomycin (Thermo Fisher Scientific; 15140122). Ascorbic acid, which is an essential factor for collagen biosynthesis^100^, was added to culture media for both cells. Prior to seeding CFs, all culture vessels were pre-coated with a gelatin-based coating solution (Cell Biologics; 6950) for one hour. Both cell lines were maintained under standard conditions of 5% CO_2_ at 37 °C and 95% humidity.

### Assessment of cell viability of fibroblasts treated with DOX

Cell viability of fibroblast cell lines treated with DOX were assessed by alamarBlue assay (Thermo Fisher Scientific; A50100) at a seeding density of 1 × 10^4^ cells per well (100 μL) in 96-well flat bottom plates. The cells were treated with a series of DOX concentrations ranging from 0 to 50 μM for 24 h. After 21 h, 10 μL of 10X alamarBlue was added to each well, and incubated for three hours. Fluorescence conversion was measured using a Synergy H1 microplate reader (Biotek) with 560 nm excitation and 590 nm emission.

### Protein expression analysis by western blot

NIH3T3 and CFs were seeded at a density of 2 × 10^5^ cells per well in 6-well plates one day prior to their treatments. Treatments were administered for 24 h, except for the SMAD2 phosphorylation study, which lasted for one hour. Cells were treated with DOX (1 μM; 0.25, 1, and 2.5 μM for CCN2 study) and 5 ng/mL TGF-β1 or the respective vehicle control, DMSO and 4 mM HCl, respectively. ECM synthesis was promoted with 100 μg/mL ascorbic acid during treatments^101^. Cellular proteins were extracted using radioimmunoprecipitation assay (RIPA) buffer (Thermo Fisher Scientific; 89901) containing Halt™ protease and phosphatase inhibitors (Thermo Fisher Scientific; 78442). Protein concentration in the cell lysates were determined using a Pierce™ BCA protein assay kit (Thermo Fisher Scientific; 23225).

Protein samples (30 μg) were prepared with 4X protein sample loading buffer (LI-COR; 928-40004), and loaded onto 4–12% NuPAGE™ Bis–Tris gels (Thermo Fisher Scientific; NP0321BOX). The proteins were transferred onto pre-activated polyvinylidene difluoride (PVDF) membranes (Thermo Fisher Scientific; IB24001) using the iBlot™ 2 gel transfer device (Thermo Fisher Scientific; IB21002) with the following stepwise conditions: (1) 20 V, 1 mA, 1 min, (2) 23 V, 1 mA, 4 min, and (3) 25 V, 1 mA, 2 min. The membranes were blocked in Intercept® (tris-buffered saline; TBS) blocking buffer (LI-COR, 927-60003) for one hour and incubated with primary antibodies (protein of interest and housekeeping primary antibody tandemly) prepared in Intercept® T20 (TBS) antibody diluent (LI-COR, 927-65001) at 4 °C overnight. After overnight incubation with the primary antibody, membranes were washed and incubated with secondary antibody for one hour then washed again. Infrared detection of antibody binding on the membranes were scanned on the LI-COR Bioscience Odyssey CLx imaging system and analyzed for densitometry using LI-COR ImageStudio software.

### Primary and secondary antibodies

Primary antibodies used in this study were: recombinant rabbit anti-cellular communication network factor 2 (CCN2) monoclonal antibody diluted to a concentration of 1:1,000 (Abcam, ab209780); rabbit anti-bone morphogenetic protein 1 (BMP1) polyclonal antibody diluted to a concentration of 1:1,000 (Thermo Fisher Scientific; PA5-103,660; RRID: AB_2852994); rabbit anti-collagen type I (COL1) polyclonal antibody diluted to a concentration of 1:1,000 (Thermo Fisher Scientific; PA1-26204; RRID: AB_2260734); rabbit anti-suppressors of mothers against decapentaplegic homolog 2 (SMAD2) polyclonal antibody diluted to a concentration of 1:1,000 (Thermo Fisher Scientific; 51–1300; RRID: AB_2533896); rabbit anti-phosphorylated SMAD2 (pSMAD2) monoclonal antibody diluted to a concentration of 1:1,000 (Thermo Fisher Scientific; MA5-15122; RRID: AB_10978317); and mouse anti-beta actin (β-actin) monoclonal antibody diluted to a concentration of 1:5,000 (Thermo Fisher Scientific; MA1-91399; RRID: AB_2273656).

For near-infrared fluorescence detection, IRDye® 800CW donkey anti-rabbit and IRDye® 680RD donkey anti-mouse secondary antibodies (LI-COR, 926-32213 and 926-68072, respectively) were used.

### Gene expression analysis by qRT-PCR

Both cell lines were seeded at a density of 2 × 10^5^ cells per well in 6 well plates. After one day, the cells were treated with 5 ng/mL TGF-β1, 100 μg/mL ascorbic acid, 1 μM DOX or their vehicle controls for 24 h. Total RNA was extracted from the cell lines using the RNeasy Mini Kit (Qiagen; 217004). RNA concentrations were measured using a NanoDrop 2000 spectrophotometer (Thermo Fisher Scientific; ND2000USCAN). Subsequently, the RT^2^ first strand kit (Qiagen; 330401) was utilized to perform reverse transcription of 100 ng RNA from each sample into cDNA, in accordance with the manufacturer's instructions. The cDNA was mixed with SYBR Green mastermix (Qiagen; 330503) for fluorescence detection using a thermocycler (LightCycler 96, Roche). We employed a TGF-β/BMP signaling pathway RT^2^ Profiler™ PCR 96 well plate containing primers specific for genes related to the TGF-β/BMP signaling cascade, RNA controls, and housekeeping genes (Qiagen; PAMM-035ZF-12; 330231). The 96-well plate was loaded with 25 µL of cDNA per well. The following thermal profile was applied: 1 cycle at 95 °C for 10 min and 45 cycles at 95 °C for 15 s, then 60 °C for one minute. A web-based tool from Qiagen, RT^2^ Profiler PCR Data Analysis, was used for differential gene expression analysis.

### Statistical analysis

The values were presented as mean ± SE. Differences between the two groups were evaluated using the Student t-test. Differences among multiple groups were evaluated using a two-way analysis of variance (ANOVA) with Dunnett’s test. The level of significance was selected to be *P* < 0.05. R statistics software was used to perform the statistical analysis.

### Supplementary Information

Supplementary Information.

## Data Availability

The authors confirm that the data supporting the findings of this study are available within the article and its supplementary materials.
