# Supplementary material for: Doxorubicin-induced modulation of TGF-β signaling cascade in mouse fibroblasts: insights into cardiotoxicity mechanisms
Source: Sci Rep. 2023 Nov 2;13:18944. doi: 10.1038/s41598-023-46216-7 (PMC10622533; doi:10.1038/s41598-023-46216-7)
Supplement: Supplementary file 1 — Supplementary Information. [file 41598_2023_46216_MOESM1_ESM.pdf]

Doxorubicin-Induced Modulation of TGF-β Signaling Cascade in Mouse Fibroblasts: Insights into Cardiotoxicity Mechanisms

Conner Patricelli [1], Parker Lehmann [2], Julia Thom Oxford [1,3,4], Xinzhu Pu [3,4*]

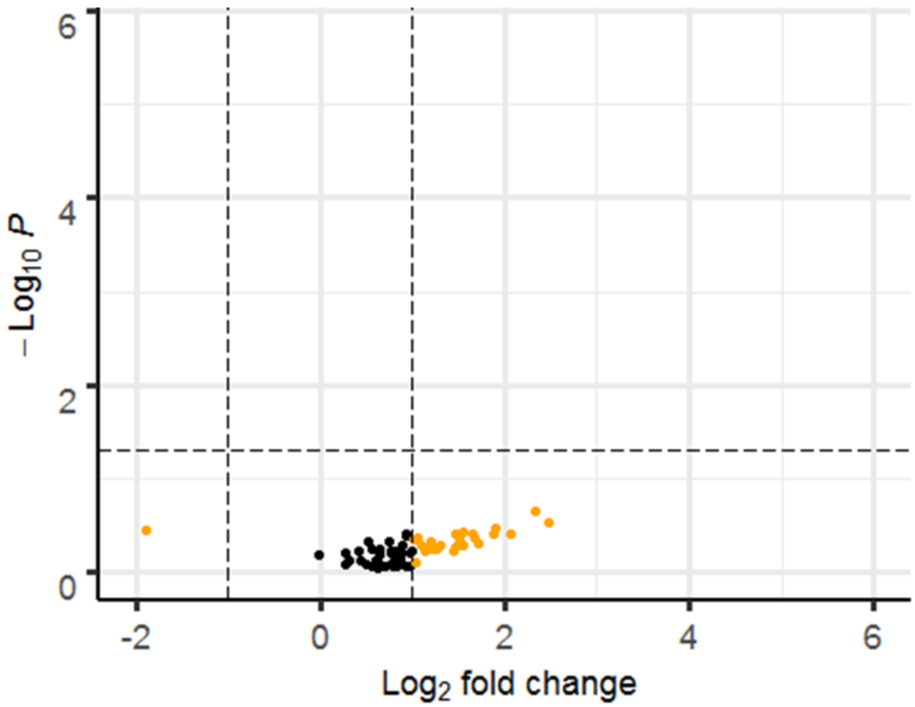

Supplement Figure 1: **No differential gene expression changes were observed in CFs.** CFs treated with 10 ng TGF-β1 and 1 µM DOX for 24 hours did not significantly alter the selected genes in the TGFβ/BMP Signaling Pathways.
